# Analyzing the temporal regulation of translation efficiency in mouse liver

**DOI:** 10.1016/j.gdata.2016.03.004

**Published:** 2016-03-18

**Authors:** Peggy Janich, Alaaddin Bulak Arpat, Violeta Castelo-Szekely, David Gatfield

**Affiliations:** a Center for Integrative Genomics, Génopode, University of Lausanne, 1015 Lausanne, Switzerland; b Vital-IT, Swiss Institute of Bioinformatics, 1015 Lausanne, Switzerland

## Abstract

Mammalian physiology and behavior follow daily rhythms that are orchestrated by endogenous timekeepers known as circadian clocks. Rhythms in transcription are considered the main mechanism to engender rhythmic gene expression, but important roles for posttranscriptional mechanisms have recently emerged as well (reviewed in Lim and Allada (2013) [1]). We have recently reported on the use of ribosome profiling (RPF-seq), a method based on the high-throughput sequencing of ribosome protected mRNA fragments, to explore the temporal regulation of translation efficiency (Janich et al., 2015 [2]). Through the comparison of around-the-clock RPF-seq and matching RNA-seq data we were able to identify 150 genes, involved in ribosome biogenesis, iron metabolism and other pathways, whose rhythmicity is generated entirely at the level of protein synthesis. The temporal transcriptome and translatome data sets from this study have been deposited in NCBI's Gene Expression Omnibus under the accession number GSE67305. Here we provide additional information on the experimental setup and on important optimization steps pertaining to the ribosome profiling technique in mouse liver and to data analysis.

**Table:** 

Specifications	
Organism/cell line/tissue	Mus musculus/liver
Sex	Male
Sequencer or array type	Illumina HiSeq 2500
Data format	Raw and processed data
Experimental factors	Livers were collected every 2 h during the 24-h daily cycle (with 2 replicate time series)
Experimental features	RNA-seq and RPF-seq were performed in parallel on the same liver lysates to identify mRNA subject to rhythmicity at the translational level
Consent	Data are publicly available at NCBI GEO
Sample source location	Lausanne, Switzerland

## 1 Direct link to deposited data

Direct link to deposited files: http://datalink.elsevier.com/midas/datalink/api/downloadfiles?items=18934-18935-18936

Direct link to deposited genomic data: http://www.ncbi.nlm.nih.gov/geo/query/acc.cgi?token=etmbssamttcnzsb&amp;acc=GSE67305

## 2 Experimental design, materials and methods

### 2.1 Experimental design

To investigate daily rhythms in translation, we recently performed ribosome profiling in mouse liver (Janich et al., 2015 [2]), which is the most commonly used peripheral organ in circadian research due to its easy dissectability, its relatively homogenous cellular composition and its abundant, high-amplitude rhythms [3]. Ribosome profiling is based on the deep sequencing of ≈ 30 nucleotide mRNA fragments that are protected by translating ribosomes upon nuclease digestion [4]. The sequence information contained in the footprints allowed us to perform transcriptome-wide, quantitative analyses of protein synthesis rhythms in mouse liver. Parallel RNA-seq data was used to quantify RNA abundance around-the-clock, allowing the identification of those genes whose rhythmicity was exclusively translational. Livers were collected at 2 h intervals around-the-clock in order to have sufficient temporal resolution for reliable rhythmicity detection. For each time point, two replicate samples were generated. Each replicate consisted of a pool of 2 individual livers.

### 2.2 Mice

Wild type C57BL/6J mice were purchased from Janvier Labs. Animal housing and experimental procedures were in agreement with the veterinary law of the Canton Vaud, Switzerland (authorization to DG: VD2376). For all experiments, mice were entrained to 12-h-light/12-h-dark cycles for 2 weeks with water and normal chow available ad libitum. Prior to organ collection, mice were anesthetized with isoflurane and sacrificed by decapitation. Mice were sacrificed at the indicated Zeitgeber times (ZT), with ZT00 corresponding to “lights on” and ZT12 to “lights off” in the animal housing facility. Livers were rapidly excised and immediately processed to lysate.

### 2.3 Lysate preparation

**Fig. 1:**
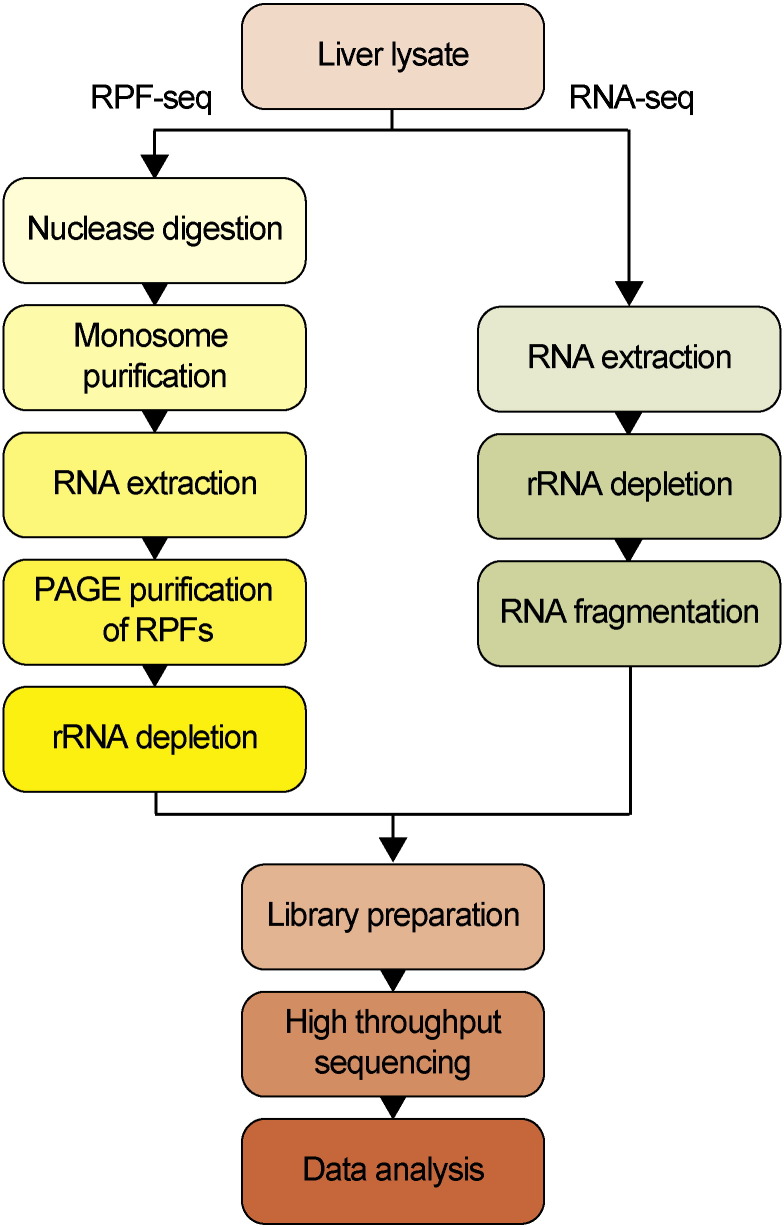
Overview of the experimental workflow used for ribosome profiling (RPF-seq) and for RNA-seq in mouse liver.

Freshly extracted liver tissue from each individual mouse was weighed and subsequently lysed with 8 strokes in a Teflon homogenizer containing 3 volumes of ice-cold lysis buffer (20 mM Tris–HCl pH 7.4, 150 mM NaCl, 5 mM MgCl_2_, 5 mM DTT, 100 μg/ml cycloheximide, 1% Triton X-100, and 0.5% sodium deoxycholate) supplemented with complete EDTA-free protease inhibitors (Roche) and 40 U/ml RNasin plus (Promega). Of note, RNasin plus inhibits RNase A, B and other RNases present in liver extracts, but not RNase I, which will be used at a later stage of the protocol to generate ribosome protected mRNA fragments (RPFs). The liver homogenates were transferred to microcentrifuge tubes and incubated for 10 min on ice. Cellular debris was pelleted by centrifugation at 1000 ×* g* for 3 min at 4 °C. The supernatant was removed, aliquoted, and snap-frozen and stored under liquid nitrogen until further processing. For absorbance measurements at 260 nm, lysates were gently thawed on ice, diluted 1:10 and 1:20 in water, the absorbance determined by Nanodrop and the average value from the two dilutions was determined. In general, the lysates ranged between 100 and 200 OD260 per 1 ml lysate. Equal amounts of lysate (OD260) from 2 mice, collected at the same time point, were pooled and diluted with lysis buffer to a final concentration of 15 OD260/100 μl. Lysates were processed separately for RPF-seq and RNA-seq (Fig. 1).

### 2.4 RNA extraction and RNA-seq library preparation

For the isolation of total (cytoplasmic) RNA, 100 μl pooled lysate was mixed with 1 ml Trizol and incubated for 5 min at room temperature. RNA was isolated using the miRNeasy kit (Qiagen) according to the manufacturer's protocol and the concentration determined by Nanodrop. Prior to library preparation, a total of 5 μg RNA was subjected to ribosomal RNA depletion (Ribo-Zero magnetic kit, Epicenter) and subsequently purified using a RNA purification kit (RNA Clean & Concentrator-5, Zymo Research). RNA-seq libraries were generated following the instructions for total RNA library preparation of the ARTseq ribosome profiling kit (Epicenter).

### 2.5 Preparation of ribosome protected mRNA fragments (RPFs)

**Fig. 2:**
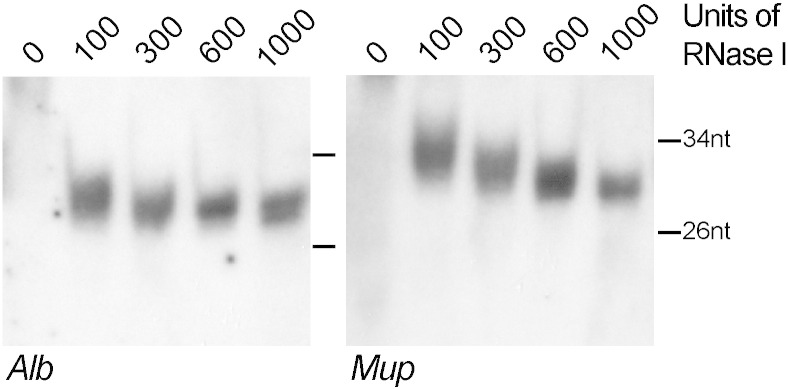
Optimization of RNase I concentration. Autoradiographs of RNase I-digested liver RPFs probed for two highly expressed liver mRNAs, albumin (Alb, probe: cgatgggcgatctcactcttgtgtgcttctc) and major urinary protein (Mup, probe: gttccttcccgtagaactagcttc). RPFs of 30 nucleotides in length were obtained when 600–1000 units of RNase I were used for digestion.

In preparation to processing the samples from the large-scale time series, we optimized the conditions for nuclease digest in order to ensure efficient and reproducible generation of RPFs of ≈ 30 nucleotides in length. To this end, liver lysates were incubated with different amounts of RNase I ranging from 0 to 1000 units (Ambion). The digested mRNA fragments were purified using Trizol extraction and analyzed by northern blot as described previously [5]. Northern blot hybridization was performed using 2 different probes recognizing two highly expressed liver mRNAs (Alb, albumin and Mup, major urinary protein). Analysis of the autoradiographs showed that the optimal concentration for obtaining mRNA fragments of 30 nucleotides in lysates prepared from mouse liver was in the range of 600 to 1000 units RNase I (Fig. 2).

**Fig. 3:**
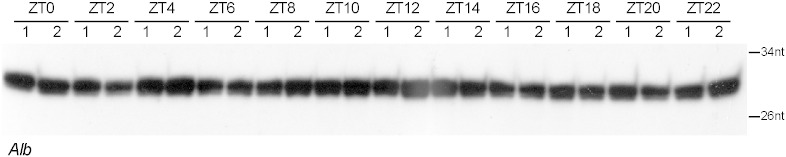
Quality control of footprints generated from time series. Autoradiograph of liver RPFs from all replicate samples of the time series experiment probed for albumin (Alb, probe: same as in Fig. 2). In all samples RPFs of 30 nucleotides in length were detected, indicating that the nuclease digestion conditions were homogenous and reproducible. ZT, Zeitgeber time.

Thus, for our time course experiment, lysates of a concentration of 15 OD260 in a volume of 100 μl were incubated with 650 units RNase I and 2.5 μl DNase I for 45 min at room temperature. After the incubation, samples were placed on ice and 8.7 μl Superasin RNase inhibitor (Ambion) were added to inactivate the RNase I enzyme. In the meantime, size exclusion spin columns (S-400, GE Healthcare Life Sciences), that would subsequently serve to purify the nuclease-generated monosomes, were prepared. To this end, spin columns were washed 3 times with 700 μl lysate buffer containing 20 U/ml Superasin in a microcentrifuge at 600 × g for 1 min. Between each washing step the matrix of the spin column was gently resuspended by vortexing. After the washing steps, lysates were applied to the matrix and the spin columns were centrifuged for 2 min at 600 ×* g*. 1 ml of Trizol was added to the flow-through and RNA was extracted using the miRNeasy kit (Qiagen) according to the instructions of the manufacturer. The concentration of the RNA was determined by Nanodrop and RNA was stored at − 80 °C. Before proceeding to library preparation, the quality of the extracted RPFs was verified for all samples of the time course experiment by northern blot analysis (Fig. 3).

### 2.6 RPF-seq library preparation and sequencing

Library preparation of RPFs was performed according to the ARTseq ribosome profiling kit (Epicenter) with the modification that the steps of ribosomal RNA depletion (Ribo-zero) and polyacrylamide gel electrophoresis (PAGE) purification were inverted. The original protocol uses 5 μg of RPF RNA as starting material, which is the maximum amount of RNA recommended in a Ribo-zero reaction. However, using this protocol we were occasionally unable to obtain sufficiently concentrated libraries for sequencing. The inversion of the 2 steps allowed us to increase the starting material up to 25 μg RPF RNA, without noticing a negative effect on the performance of the Ribo-zero depletion. In our case, the RNA was first separated on a 15% urea-polyacrylamide gel and stained with SYBR-Gold (Invitrogen). Gel pieces between 26 and 34 nucleotides were excised, the RNA extracted, purified and only then used for ribosomal RNA depletion. After the Ribo-zero reaction, the RNA was purified using a RNA purification kit (RNA Clean & Concentrator-5, Zymo Research) and immediately subjected to end-repair and 3′ adaptor ligation according to the instructions of the ARTseq protocol. Reverse transcription, cDNA purification and circularization were done as described in the ARTseq protocol. During the last step of library preparation, i.e. the PCR amplification, 5 μl out of the 20 μl circularized cDNA product was used as template in the PCR reaction. The quality of the final library was assessed by analyzing 5 μl of the PCR reaction on an 8% native PAGE, stained with SYBR Gold. For some libraries, only a faint band was visible, and we then performed up to 3 additional PCR amplifications as described above in order to have sufficient material. In a final step, the identical PCR reactions were pooled and loaded on an 8% native PAGE. The library running at ≈ 150 bp was gel-purified, precipitated with ammonium acetate and resuspended in water. The concentration and the quality of both RPF-seq and RNA-seq libraries were determined by Qubit (Thermo Fisher) and BioAnalyzer (Agilent). Up to 6 libraries were multiplexed and subjected to 100 cycles of single-end sequencing on an Illumina HiSeq2500 (Illumina).

### 2.7 Data processing and analysis

Here, we briefly outline the first steps of data analysis. More detailed information on data processing and analysis can be found in the supplementary information of the original publication [2].

In a first step, sequencing reads were de-multiplexed, adapter-trimmed and size-filtered (lengths of 26–35 nucleotides for RPF-seq and 21–60 nucleotides for RNA-seq). Next, we removed from the filtered reads sequentially the mouse and human ribosomal rRNA, mouse mt-tRNA, mouse tRNA, and then finally aligned the reads to mouse cDNA (Ensembl release 75) and genomic sequences (GRCm38.p2), using bowtie version 2.2.1 and Tophat v2.0.11, respectively. In parallel, a database of expressed transcripts was generated from RNA-seq reads (cufflinks v2.2.1) and used for subsequent analyses. Read quantification was done per gene for each annotation feature (5′ UTR, CDS, 3′ UTR). Read counts were normalized with the upper quantile method of R package edgeR v3.4.2 and RPKM values were calculated as the number of counted reads per 1000 mapped and counted bases per geometric mean of normalized read counts per million (RPKM). Translation efficiencies were calculated as the ratio of RPF-RPKM/mRNA RPKM. The Babel framework was used to assess significant changes in translational regulation [6]. We used a mixed model approach for rhythmicity detection (sigmoid and sinusoidal curve fittings). Rhythmic transcripts were defined as those with a peak-to-through amplitude > 1.5 and an FDR for rhythmicity detection < 0.05.

## 3 Discussion

We describe here two temporal data sets in mouse liver composed of transcriptome (RNA-seq) and translatome profiling (RPF-seq) with 2-h resolution around the 24-h daily cycle. Rhythmic RNA abundance and rhythmic footprints affected a similar proportion (17%) of the total 10′800 genes detected in both RNA-seq and RPF-seq datasets. The majority of these rhythmic events (≈ 1200 genes) oscillated both at the mRNA abundance and the translation level. However, we also identified ≈ 150 genes that showed no daily changes in mRNA abundance but rhythms in translation. These genes are involved in ribosome biogenesis, translation regulation, iron homeostasis and other pathways. Future studies will aim at uncovering the precise mechanisms that engender rhythmicity at the translational level, thus adding a new level of understanding to posttranscriptional control of rhythmic gene expression (Lim and Allada (2013) [1]). In summary, the around-the-clock RPF-seq and matching RNA-seq datasets provide valuable information about the role of translation in generating rhythmic gene outputs, but the data will likely be of high interest to researchers outside the circadian field as well.

## Conflict of interest

The authors declare no conflicts of interests.
